# Supplementary material for: The interactome of intact mitochondria by cross-linking mass spectrometry provides evidence for coexisting respiratory supercomplexes
Source: Mol Cell Proteomics. 2017 Dec 8;17(2):216–32. doi: 10.1074/mcp.RA117.000470 (PMC5795388; doi:10.1074/mcp.RA117.000470)
Supplement: Supplemental Data [file supp_RA117.000470_133922_0_supp_23978_fzffwf.zip › spectra_annotation/mito_DR_spectra_annotation/312-1-8-1-4-1.pdf]

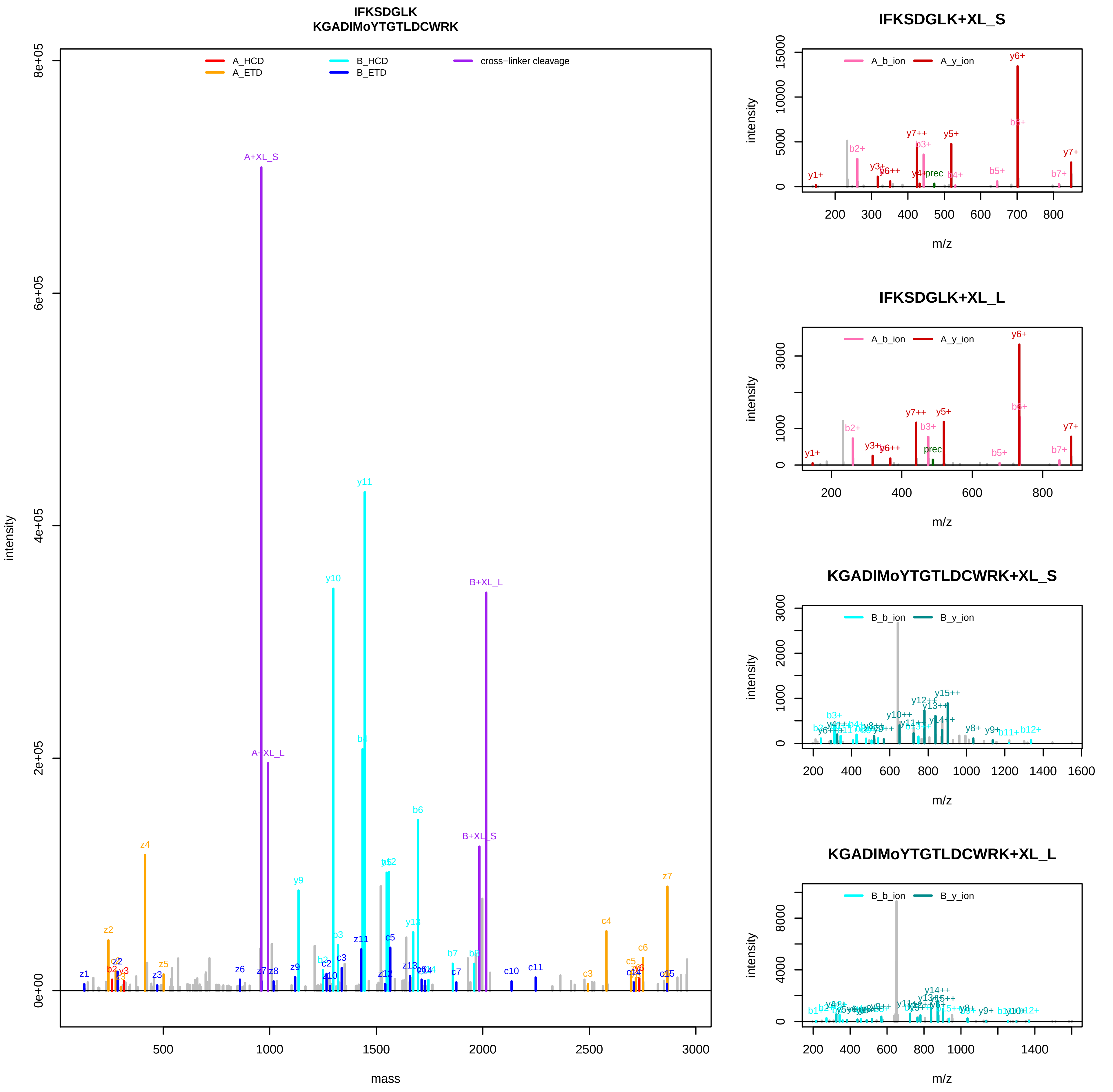

IFKSDGLK
KGADIMoYTGTLDCWRK
A_HCD
A_ETD
B_HCD
B_ETD
cross−linker cleavage
intensity
mass
0e+00
2e+05
4e+05
6e+05
8e+05
500
1000
1500
2000
2500
3000
A+XL_S
A+XL_L
B+XL_L
B+XL_S
y11
y10
y9
b6
b4
y13
z4
z2
z7
c4
c6
c5
z1
z3
z5
z6
z7
z8
z9
z11
z12
z13
c2
c3
c5
c7
c10
c11
c14
c15
b2
y3
IFKSDGLK+XL_S
IFKSDGLK+XL_L
KGADIMoYTGTLDCWRK+XL_S
KGADIMoYTGTLDCWRK+XL_L
A_b_ion
A_y_ion
B_b_ion
B_y_ion
m/z
y1+
b2+
y3+
y6++
y4+
prec
y7++
b3+
y5+
b4+
b5+
b6+
y6+
b7+
y7+
b3+
y10++
y12++
y13++
y15++
y14++
y11+
y8+
y9+
b11+
b12+
